# Bleaching Teeth

**Published:** 1881-07

**Authors:** James Truman

**Affiliations:** Philadelphia, Pa.


[TO BE CONTINUED.]

---

### ARTICLE III.

### *Bleaching Teeth.*—(Continued.)

BY JAMES TRUMAN, D. D. S., PHILADELPHIA, PA.

The practice formerly adopted was rather of a palliative character than of any positive bleaching treatment; indeed but little attention was given it at all. Such has been the singular indifference in regard to it that works on operative dentistry either treated it as of small moment or passed it by as unworthy of any notice. The result is that students in quest of information must look elsewhere than to the text-books for the information desired. While it is true that in the older sections of the country, where the people have for a long period been educated properly in regaad to the care of the teeth, this difficulty of discoloration is not as frequently presented as formerly; but it is evident, from the frequent demands for information, that such is not the case everywhere. That the indifference is in part due to the failure of the various processes that have from time to time been recommended, must be conceded, but it would be well to remember that failure may be the result of a want of manipulative dexterity, of patience in attending to all the details, and, above all, a lack of care in the preliminary treatment. There is, doubtless, much in all the processes that have been recommended. Even the old ones, that aimed only to change the color by mechanical displacement, such as the use of starch, magnesia, etc., had a certain degree of merit. The advance was great when chlorinated lime was adopted, though the full value of this material was not appreciated until a later period.

The possibility of bleaching tooth-tissue is based on the fact that the dentine is permeated throughout by the so-called tubuli. Each of these microscopic conduits is traversed throughout its entire extent by a soft tissue. This much has been positively demonstrated. It has been also demonstrated that coloring-matter can be carried, under ordinary conditions, nearly to the final distribution of the minute ramifications of these tubuli. This is an important point for without this vascularity, bleaching would be impossible. With it the possibility exists of extending the whitening process to the peripheral border of the dentine, or, in other words, to its union with the enamel in the crown or cementum in the root.

It may be argued, and with considerable force, that if the tubuli are the channels of organic material, this, necessarily, will decompose and become a source of discoloration. This I believe to be true, and, in some instances, it may be the cause of re-discoloration. But this latter difficulty is, in my experience, so exceptional that it furnishes no basis for an argument against the treatment as a whole.

The diameter of the tubuli is so minute—always decreasing in size until lost in final distribution—that any agent used must require considerable time before it can penetrate to the minutest ramifications. This is demonstrated by attempts to color dentine through the pulp-canal. It is an exceedingly slow process, hence, any attempt, in my judgment, to bleach rapidly, must prove a partial failure. The change, if change be made at all, is simply on the walls of the canal, and cannot penetrate to any depth of tissue. If the discoloration is superficial this mode will be effectual, but not otherwise. So difficult is it to penetrate the tissue in a certain class of teeth with large crowns that I have repeatedly spent several months in the vain effort to bleach at the neck. Such cases are rare, and they were taken with the view of experiment. It must be apparent that any agent used should have a penetrating as well as a bleaching power, and, at the same time, must not have a deleterious influence on tooth-structure.

Color can be changed by several of the acids, notably oxalic and nitric. The former destroys the color and the latter changes dark blue to a yellowish tinge; but, as both of these are destructive, they should never be used except in connection with an antacid. My own experience with the first-named has been wholly unsatisfactory, though it will be observed in the details of Dr. Huey's mode that he uses it in combination with chlorinated lime, and with excellent results. Experience does not warrant me in giving an opinion as to the merits of its use in this connection.

Chlorine free, or in some of its combinations, has been the main reliance for bleaching, and that it has been the most effectual has been demonstrated so thoroughly that any attempt now to assert to the contrary must be regarded as the result of defective manipulation or absolute ignorance. It has great penetrating power, and is a thorough bleacher; it is readily applied, and is harmless. It remains therefore, only to consider the most feasible, and, at the same time, most effectual mode for its administration.

As has been already remarked, chlorinated lime was early used, and had a partial successs. This was due mainly to the fact there is a certain amount of acid in the cavity of the tooth,—the result of decomposition. This, brought in contact with the lime, sets free a small portion of the chlorine, but the amount is too small to be effective. Chlorine is liberated by all the acids,—very rapidly by tartaric and sulphuric, and in less degree by acetic. The idea previously advanced being conceded that the penetration of the tubuli must necessarily be slow, it follows that a sudden presentation must fail, for want of time, to penetrate the tubuli; hence the process, so warmly recommended by some, of bleaching by direct contact with chlorine generated in a bottle could only be partially successful.

The use of chlorine in this way cannot be commended, for while it bleaches superficially, it has the objection before mentioned of non-penetrating power, and has another, and a very serious one, of being annoying to both patient and operator. What is wanted is slow action,—the slower the better,—and this process does not meet this demand. This was so clearly manifested in my earlier experiences that I early abandoned all powerful action, and reduced the acids finally adopted to the strength of about a ten per cent. solution. This was subsequently increased until, at times, I used acetic acid at full strength; but I am still of the opinion that weak solutions are the most effective.

It will be apparent then that, having thrown out all the agents mentioned, either from their ineffectiveness or deleterious character, the chlorine compounds alone remain. Of these, experience has demonstrated the chlorinated lime to be the most effective.

The difficulty attending the use of this material is to be found in the fact that it is rare to find the article good. Most of that sold in the shops is absolutely worthless. So difficult has this been that one very successful operator in this specialty prepared for a time his own, but this was found unsatisfactory in a privite laboratory. The best plan is to procure it from first hands, having it carefully bottled, and always kept so, as contact with the atmosphere destroys its value.

A variety of tests are used in chloromety to ascertain the exact amount of chlorine present in a given quantity of bleaching-powder, but as exactness is unnecessary for the object aimed at in this treatment, the operator can, if it is found requisite to test at all, make use of the crude but effectual indigo test. To a solution of indigo in a test-tube, add a small amount of chlorinated lime; to this add strong acid. The rapidity of the change of color will indicate the relative amount of chlorine in combination with the lime. If there is very little change of color, or the bleaching proceeds slowly, the lime should be discarded as unfit for use. This is more important than it may seem to be; for if the operator is not sure of the quality of his lime, the whole process will be uncertain, and may possibly result in failure. Good chlorinated lime is in the form of a dry powder. In this condition it is exceedingly difficult to insert properly in a cavity.

To accomplish this, proper instruments must be kept on hand and used only for this operation. The best forms are those devised by Dr. Huey, the handles being of socket-form, with bits of gold or platinum. The latter materials are used to avoid the deleterious action of acids. A very good instrument is the Holmes platinum-point plugger, for oxychloride fillings, No. 1. With an additional point, smaller, for packing in the canal, and a bit formed like a hoe-shaped excavator, the outfit will be complete. These cover all the essential ideas in Dr. Huey's instruments. It must be borne in mind that *no steel instrument can be brought in contact with the tooth in this operation.* The neglect of this plain requirement has caused most extensive discoloration. Instruments made of hard wood or ivory will be found nearly equally as good as those made of gold or platinum, and they have the merit of being less expensive.

The next point to be considered is the insertion of the material, which, simple as it seems, requires considerable dexterity. The tooth should be prepared by thorough cleansing of all foreign matter, and then encircled with the rubber-dam. With the lime and acid, have also the gutta-percha ready prepared for immediate application. The difficult point is to bring the acid in connection with the lime, so that, while it may accomplish the work effectually, there may be no loss of chlorine. I have tried several methods, but I think that of dipping the instrument in the weak acid solution, then in the lime, and inserting this rapidly in the cavity, is the best. It has the objection that it requires very rapid manipulation. Another plan has been to use distilled water to make a paste, and when sufficient has been inserted, apply a stronger acid by means of cotton wrapped around one of the platinum-points. The objections to this are the stronger acid and the uncertainty attending it. I, therefore, prefer the first mode. When the cavity is sufficiently full, it should be immediately sealed with gutta-percha and allowed to rest for several days. On the return of the patient the cavity and canal should be thoroughly cleansed with distilled water. If the bleaching has not gone far enough,—which will be indicated by the color of the tooth,—a second application must be made, and this must be repeated until a satisfactory result is attained. I have not found it necessary to make more than two applications a week.

The question has been frequently asked, and probably will be again, what effect has this on tooth-structure? From my observations, none at all. If the acid is not in excess,—and it should not be,—there can be nothing deleterious in it, as the affinity of the acid for the lime destroys at once its power to act on the dentine. I have carried the operation, as before remarked, to a period of several months, and have never observed the slightest change in tissue, and I have treated teeth with the mer st film of dentine on the labial surfaces, and with equally good results.

The immediate bleaching effect will be observed in the lower third of the tooth where the dentine is thinnest. In the majority of cases this will be effected by one application; the greatest difficulty will be found at the gingival border. Here the dentine is very thick, and, as before stated, it will be very slow work, and end, in some cases, in failure to restore normal color. As this does not, however, materially affect the general appearance, I do not regard it as essential to carry the process to a length of time necessary to effect complete bleaching.

The great objection made to any effort of this kind is that the tooth "will re-discolor." This point has already been alluded to. My own experience is that there is either no return, or in so slight a degree as to be no cause of uneasiness. I have already intimated that this re-discoloration may proceed from the organic matter in the tubuli and the minute branches. If this idea be the true solution, it renders the necessity for slow and thorough bleaching all the more imperative, that the process may be carried to the minutest ramifications; but I must regard it as proceeding in most cases, from defective manipulation. Either the root *has not been bleached* or the pulp-chamber in the crown *has not been thoroughly excavated.* It is folly to imagine that the bleaching of the crown will be sufficient. The intimate connection between the tubuli in the root and those in the crown renders this course imperatively necessary; hence, the very great importance of filling only the upper third of the canal.

Dr. Robert Huey has for several years been interested in practical and experimental work with this process, modified by experience, and as his method differs from my own in some of the details, I have requested a statement which he kindly furnishes, as follows:

"The tooth must be thoroughly cleared of all decayed matter throughout the entire extent of the canal and pulp-chamber. Follow this with a wash of aqua ammoniæ, as it has been found that any portion of oily matter interferes with the action of the chlorine. Place the rubber-dam on the tooth and fill the upper part of the canal contiguous to the foramen. Prepare a solution of oxalic acid after the following formula:

℞.—Acidi oxalici, gr. x.
Aquæ destillatæ, f℥j.—M.

Have ready the very best chlorinated lime that can be procured.

"I commence the bleaching process by dipping one of the gold instruments in the acid solution, and again in the lime, and carry this as far into the tooth as possible, continuing until the cavity is full. In the course of five minutes this is removed, and the cavity refilled with fresh material, and so continue three or four times in the course

3

of an hour. The largest proportion of cases I have had under treatment have been finished at one sitting. I prefer to use oxalic acid to liberate the chlorine, as the action is more rapid than by either acetic or tartaric, and seems to be more effectual in very difficult cases. If it is necessary to use a temporary stopping after bleaching, I prefer a cement filling to one of gutta-percha. The objection to the latter is that after its use, in some cases, re-discoloration has followed, caused by the admission, I believe, of moisture. The cement filling should not be inserted until after precautions have been taken to dry all the parts of the cavity and canal. For this purpose the hot-air syringe is absolutely essential. Any moisture left in is injurious. The cement filling should have a putty-like consistence. When it is necessary to keep the bleaching material in for a longer period than one sitting, gutta-percha must be employed to seal up the cavity."—*Cosmos.*

